# A new patient registry for Chagas disease

**DOI:** 10.1371/journal.pntd.0008418

**Published:** 2020-10-01

**Authors:** Peter Hotez, Maria Elena Bottazzi, Nathalie Strub-Wourgaft, Sergio Sosa-Estani, Faustino Torrico, Leire Pajín, Marcelo Abril, Javier Sancho

**Affiliations:** 1 Global Chagas Disease Coalition, Barcelona, Spain; 2 Texas Children’s Hospital Center for Vaccine Development, National School of Tropical Medicine, Baylor College of Medicine, Houston, Texas, United States of America; 3 Department of Biology, Baylor University, Waco, Texas, United States of America; 4 Hagler Institute for Advanced Studies at Texas A&M University, College Station, Texas, United States of America; 5 Scowcroft Institute of International Studies, Bush School of Government and Public Service, Texas A&M University, College Station, Texas, United States of America; 6 James A Baker III Institute of Public Policy, Rice University, Houston, Texas, United States; 7 Drugs for Neglected Diseases Initiative (DNDi), Geneva, Switzerland; 8 Fundación Ciencias y Estudios Aplicados para el Desarrollo y Salud Medio Ambiente (CEADES), Cochabamba, Bolivia; 9 Barcelona Institute for Global Health (ISGlobal), Hospital Clínic, University of Barcelona, Barcelona, Spain; 10 Fundación Mundo Sano, Madrid, Spain; University of Texas at El Paso, UNITED STATES

There is urgency to establish a new and comprehensive patient registry for the millions of people who live in vulnerable social conditions with Chagas disease.

First established in the early 1990s, today, national cancer registries in both the United States and Europe contribute to our understanding of the natural history of cancer and play an important role in improving patient treatment and prevention outcomes. In the US, the National Program of Cancer Registries (NPCR) was created by Congress in 1992, covering 46 states (in addition to the District of Columbia, Puerto Rico, and US Pacific and Virgin Islands), and administered by the US Centers for Disease Control and Prevention (CDC) [1, 2]. In addition, the US National Cancer Institute (NCI) administers a second registry known as the Surveillance, Epidemiology, and End Results (SEER). Both registries collect information for the entire US population. According to the CDC, this extensive coverage at the national level “enables researchers, clinicians, policy makers, public health professionals, and members of the public to monitor the burden of cancer, evaluate the successes of programs, and identify additional needs for cancer prevention and control efforts at national, state, and local levels” [1]. The registries also provide high quality data to evaluate shifting trends in cancer over time, geographic variations in local cancer incidence, and specific at risk populations, especially among ethnic groups [3]. Across Europe, a European Network of Cancer Registries (ENCR) performs similar functions [4]. It was preceded by the longitudinal Framingham Heart cohort study, which was initiated in the US in 1948 and identifed the risk factors associated with the development of cardiovascular coronary disease, which were relevant to develop prevention programs [5].

Today, disease registries are also not restricted to cancer. For instance, the US National Institutes of Health either oversees or maintains registries for a variety of chronic illnesses, including Alzheimer’s disease, cerebral palsy, congenital heart and muscle diseases, cystic fibrosis, drug induced liver disease, lupus, and several rare diseases, among others [6].

Another case to highlight among infectious diseases is HIV-AIDS surveillance [7, 8]. This started by reporting AIDS and allowed us to establish clinical guidelines and the initial elements of disease epidemiology. Later, following the implementation of antiretroviral drug therapy, it facilitated HIV-AIDS disease notification and, ultimately, a better understanding of virus transmission dynamics and disease burden. It also shifted the public health system in many areas to manage the establishment of disease testing and treatment programs.

Along these lines, there is also a rationale for establishing a registry for selected neglected tropical diseases (NTDs). Many NTDs are chronic and debilitating conditions that resemble noncommunicable diseases or chronic infections such as HIV-AIDS [9]. Several NTDs also have uncertain treatment outcomes, while, in many cases, large patient populations lack access to interventions because they are disproportionately comprised of people living in extreme poverty or underserved indigenous populations. For these reasons, some NTDs might also be suitable for a registry.

We feel there is an urgency to establish a registry for the 6 to 7 million people living with Chagas disease [10]. Following infection through the bite of a triatomine (kissing bug), oral ingestion, or vertical or blood transfusion, the *Trypanosoma cruzi* protozoan parasite can cause acute Chagas disease. However, this initial phase of the infection is often undetected and following by a period of latency that can last years or even decades, a condition known as the indeterminate phase. Very little is known about the progression. A majority of patients never progress to symptoms, but up to one-third can enter the determinate phase and develop chagasic heart disease [11], also known as chronic chagasic cardiomyopathy (CCC), and/or severe chagasic gastrointestinal disease characterized by megacolon and/or megaesophaus. Less than 1% to 2% of patients have access to etiological treatment, and, when performed in the chronic phase, there is no definitive understanding or demonstration on its long-term impact.

A Chagas disease registry could become a useful and vital tool for answering some key questions regarding the epidemiology, disease burden, and natural history and treatment of Chagas disease. For instance, what is the best estimation of people infected (up today very criticized), why do some patients retain their indeterminate status and remain asymptomatic across their life span (whereas others progress to CCC and or gastrointestinal disease), and what are the risk factors associated with disease progression? Is there a specific patient population with a greater likelihood of remaining indeterminate versus entering the determinate phase? Alternatively, is there a specific geographic region of the Americas or mode of transmission that triggers determinate status? What is the trend over time (by decades) after interventions for primary prevention?

Still another critical role for a Chagas disease patient registry is its potential role in patient care and treatment. The results of the randomized BENznidazole Evaluation For Interrupting Trypanosomiasis (BENEFIT) study found that benznidazole when administered after the onset of CCC does not alter the progression of heart disease or improve mortality [12]. It also, unexpectedly, showed that almost one-fifth of patients with CCC die within five years, a higher mortality than previously shown [12]. A Chagas disease registry would help to clarify the true effects of antiparasitic treatment and length of treatment required as well as the added benefits of new adjunct therapies to improve heart function, such as Parachute-HF Trial [13]. It would help to evaluate criteria and successes in heart transplantation and provide a framework to determine the success of expected extensive treatment program with the current drugs and new Chagas therapies in development, such as fexinidazole or new immunotherapies [14]. A registry would also be of great help in the monitoring, diagnosis, and treatment of the children of infected women [15]. A Chagas disease patient registry could be paired with serum chemistries in order to aid the development of biomarkers to predict downstream treatment successes versus progression of disease [16]. As in others NTDs, there are limitations in the availability of evidence-based guidelines for Chagas disease or, in some cases, insufficient evidence to support some of the guidelines even when they are available[17, 18]. In some cases, there is also a lack of minimum standardization between and within the different countries, which created even greater obstacles for an integrated global response [19].

A Chagas disease patient registry would provide a needed platform to assess patient care and quality improvement, while simultaneously raising awareness regarding the absence of access to essential medicine for patients living with the disease. By some estimates, only 1% to 2% of patients living with Chagas disease are reported to national health ministries or have access to diagnosis and treatment [20–23]. Even fewer likely have access to a cardiologist. Establishing a Chagas disease patient registry could create a new practice paradigm and stimulate clinicians to both report cases and enroll them in treatment protocols or clinical trials.

Currently, an Infectious Diseases Data Observatory (IDDO) based at Oxford University to assemble clinical, laboratory, and epidemiology data for Ebola, leishmaniasis, and malaria (https://www.iddo.org/) is in place and could potentially be extended to include Chagas disease. However, to date, most of the information collected through IDDO is retrospective and would be limited in its ability to address the prospective studies required to address the major patient critical questions outlined above. Ultimately, the global resources are available to establish a registry. Today, more than 90% of patients living with Chagas disease live in the largest economies in the Western Hemisphere: Argentina, Brazil, Mexico, and the UUS [24], while substantial numbers live in Spain and elsewhere in Europe [25] (Fig 1).

**Fig 1:**
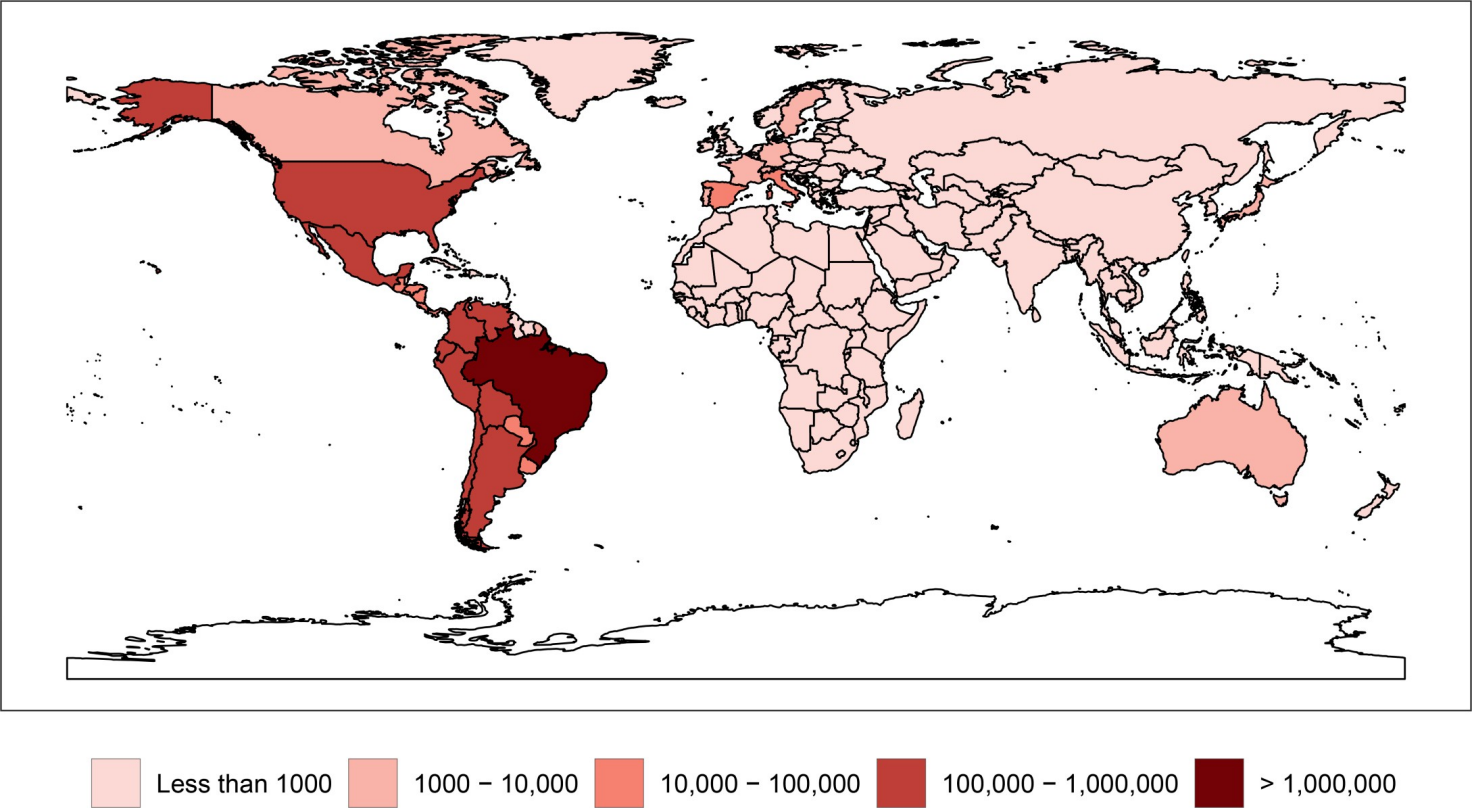
Global prevalence of Chagas disease, 2017. Original figure made with R Studio (https://cran.r-project.org/web/packages/rnaturalearth/README.html), based on data from Global Burden of Disease, http://ghdx.healthdata.org/. Permalink to specific data search: http://ghdx.healthdata.org/gbd-results-tool?params = gbd-api-2017-permalink/673265504c0639ae674897b906bd1ec4.

We therefore must expand advocacy efforts to mobilize these resources and address what has become a leading health disparity among the poor in the Americas. There are several candidate health organizations for potentially hosting a Chagas disease patient registry. They include (1) public institutions, such as the World Health Organization, promoting a sentinel network [26] or its regional affiliate in the Americas (Pan American Health Organization), (2) a major health research institution located in an endemic nation, such as Brazil’s Oswaldo Cruz Foundation (FIOCRUZ), Mexico’s National Institute of Public Health (INSP), and Argentina’s National Laboratories and Health Institutes Administration (ANLIS), or (3) one or more members of the Global Chagas Disease Coalition. The Ministry of Health of Brazil has recently taken an important step in this direction, becoming the first country in the world to include chronic Chagas disease throughout the national territory in the National List of Compulsory Notification of diseases. The rules and procedures required for notification will be defined within 90 days of the publication of the law, dated February 17, 2020 [27]. While we aspire to establish a global registry, we recognize the ambitious nature of this pursuit, especially during a COVID19 pandemic. Therefore, it might begin more modestly, through selected national registries, depending on prioritization and health system strengths.

Ultimately, a registry represents a low-cost, yet paradigm-shifting approach to enhance access to essential medicines and healthcare for the poorest people in the Americas and globally. A registry on Chagas disease should be seen as a tool for public health and will need to be sustained for a long-time (10 to 20 years). It should, therefore, command a consortium of funding institutions to ensure sustainable commitment. It comprises an essential piece of the goals and targets of the London Declaration for NTDs and, more recently, manifested by the research community through the Santa Cruz Letter [28] and PAHO summit [29, 30]. Appended is a list of key thought leaders and researchers committed to the elimination of Chagas disease (S1 Appendix).

## Supporting information

S1 Appendix List of key thought leaders and researchers committed to the elimination of Chagas disease. (DOCX)
