# Supplementary material for: A new patient registry for Chagas disease
Source: PLoS Negl Trop Dis. 2020 Oct 1;14(10):e0008418. doi: 10.1371/journal.pntd.0008418 (PMC7529299; doi:10.1371/journal.pntd.0008418)
Supplement: S1 Appendix — (DOCX) [file pntd.0008418.s001.docx]

**Appendix 1**

**Endorsement signatures:**

Joaquim Gascon^1,2^

Maria Jesús Pinazo^1,2^

Sheba Meymandi^1,3^

Pierre Buekens^1,4^

Belkisyole Alarcon^1,5^

Oscar Noya^1,6^

Andrea Angheben^1,7^

Mario J. Grijalva^1,8,9^

Ana Lemos^10^

Marta Cañas ^11^

David Moore^13^

Tania Araujo^14^

Andréa Silvestre de Sousa^14,15^

^1^Global Chagas Disease Coalition, Barcelona, Spain (Coordinator)

^2^Barcelona Institute for Global Health (ISGlobal), Hospital Clínic—University of Barcelona

^3^Olive View-UCLA Medical Center, Los Angeles, California, USA

^4^Center for Emerging Reproductive and Perinatal Epidemiology (CERPE) . Department of Epidemiology. School of Public Health and Tropical Medicine Tulane University

^5^Instituto de Medicina Tropical, Faculty of Medicine, Universidad Central de Venezuela in Caracas, Venezuela

^6^Centro para Estudios Sobre Malaria, Instituto de Altos Estudios "Dr. Arnoldo Gabaldón", Minister Of Health. Caracas, Venezuela.

^7^Department of Infectious - Tropical Diseases and Microbiology, IRCCS Sacro Cuore Hospital, Negrar di Valpolicella (Verona), Italy.

^8^Infectious and Tropical Disease Institute (Director). Biomedical Sciences Department (Professor). Heritage College of Osteopathic Medicine. Ohio University

^9^Center for Research on Health in Latin America (Director). School of Biological Sciences. Pontifical Catholic University of Ecuador, Quito

^10^Médicos Sem Fronteiras Brasil / Médicins Sans Frontières Brasil  (General Director)

^11^Médicos Sin Fronteras España // Médicins Sans Frontières Spain (General Director)

^12^Fundación Ciencias y Estudios Aplicados para el Desarrollo y Salud Medio Ambiente (CEADES)_Bolivia

^13^UK Chagas Hub. The London Centre for Neglected Tropical Disease Research (LCNTDR)

^14^Oswaldo Cruz Foundation, Evandro Chagas National Institute of Infectious Diseases, Rio de Janeiro, Rio de Janeiro, Brazil

^15^Federal University of Rio de Janeiro, Department of Internal Medicine, Rio de Janeiro, Rio de Janeiro, Brazil
